# Positive and negative impacts of nonspecific sites during target location by a sequence-specific DNA-binding protein: origin of the optimal search at physiological ionic strength

**DOI:** 10.1093/nar/gku418

**Published:** 2014-05-16

**Authors:** Alexandre Esadze, Catherine A. Kemme, Anatoly B. Kolomeisky, Junji Iwahara

**Affiliations:** 1 Department of Biochemistry and Molecular Biology, Sealy Center for Structural Biology and Molecular Biophysics, University of Texas Medical Branch, Galveston, TX 77555, USA; 2 Department of Chemistry and Center for Theoretical Biological Physics, Rice University, Houston, TX 77005, USA

## Abstract

The inducible transcription factor Egr-1, which recognizes a 9-bp target DNA sequence via three zinc-finger domains, rapidly activates particular genes upon cellular stimuli such as neuronal signals and vascular stresses. Here, using the stopped-flow fluorescence method, we measured the target search kinetics of the Egr-1 zinc-finger protein at various ionic strengths between 40 and 400 mM KCl and found the most efficient search at 150 mM KCl. We further investigated the kinetics of intersegment transfer, dissociation, and sliding of this protein on DNA at distinct concentrations of KCl. Our data suggest that Egr-1's kinetic properties are well suited for efficient scanning of chromosomal DNA *in vivo*. Based on a newly developed theory, we analyzed the origin of the optimal search efficiency at physiological ionic strength. Target association is accelerated by nonspecific binding to nearby sites and subsequent sliding to the target as well as by intersegment transfer. Although these effects are stronger at lower ionic strengths, such conditions also favor trapping of the protein at distant nonspecific sites, decelerating the target association. Our data demonstrate that Egr-1 achieves the optimal search at physiological ionic strength through a compromise between the positive and negative impacts of nonspecific interactions with DNA.

## INTRODUCTION

To perform their function, most transcription factors and DNA repair/modifying enzymes must stochastically scan DNA and locate their specific target sites in the vast presence of nonspecific but structurally similar sites. Nonspecific interactions with DNA have both positive and negative impacts on the kinetics of the target search by these proteins. Nonspecific sites near the target serve as an antenna that accelerates target location by the proteins via nonspecific association followed by sliding on DNA (1–10). At the same time, nonspecific sites distant from the target can trap the proteins and decelerate the target search process (6,11). This trapping effect can be particularly strong in the nucleus, where the DNA density is extremely high (∼100 mg/ml) (12). Although ∼80% of chromosomal DNA is occupied by nucleosomes, the total concentration of accessible DNA segments between nucleosomes (average linker length ∼56 bp) (13) is estimated to be ∼0.5 mM. Because this concentration is far higher than the typical apparent affinities of sequence-specific DNA-binding proteins for nonspecific DNA, it is qualitatively obvious that nonspecific DNA effectively traps the proteins and decelerates their target search process. However, the attenuation of search efficiency due to the trapping effect and its relationship to the mechanisms of facilitated target location by these proteins are not well understood quantitatively.

Here we address this issue for the inducible transcription factor Egr-1 (also known as Zif268), which recognizes the 9-base-pair (bp) target sequences GCG(T/G)GGGCG via three zinc-finger domains (14,15). In the brain, Egr-1 is induced by synaptic signals and activates genes for long-term memory formation and consolidation (16,17). In the cardiovascular system, Egr-1 serves as a stress-inducible transcription factor that activates the genes to initiate defense against vascular stress and injury (18,19). Within its short lifetime (∼0.5–1 h) (18), Egr-1 rapidly regulates the target genes, allowing the cells to quickly respond to stimuli. It is therefore important to understand how Egr-1 efficiently scans DNA. Our previous biophysical studies have revealed important features of DNA scanning by Egr-1 (20–23). The nuclear magnetic resonance (NMR) and stopped-flow fluorescence studies demonstrated extremely efficient intersegment transfer of Egr-1 between nonspecific DNA duplexes (20,23). The NMR study showed that Egr-1 scans DNA via two conformationally different states termed the search and recognition modes (23). In the search mode, one zinc finger is locally dissociated from DNA while the other two zinc fingers remain bound to DNA. Our computational and experimental studies have suggested that the search mode facilitates intersegment transfer via intermediates in which a protein molecule transiently bridges two DNA duplexes (21,23).

In the current study, we measure the target search kinetics as a function of ionic strength and demonstrate that Egr-1 exhibits its optimal search efficiency at physiological ionic strength. Varying the ionic strength influences the antenna and trapping effects in completely different manners and therefore allows us to quantitatively assess the interplay between these effects in the target DNA search process. We also introduce a new theoretical method that explicitly investigates the effect of non-specific interactions and intersegment transfer. Analytical expressions for the target search kinetics in terms of the enhancement factors of the antenna effect and intersegment transfer and the attenuation factor of the trapping effect are obtained. Our experimental data and the newly developed theory shed light on Egr-1's properties that are well suited for efficient scanning of DNA in the nucleus.

## MATERIALS AND METHODS

### Protein and DNA

In this study, we used a protein construct of the Egr-1 DNA-binding domain comprising three zinc fingers (human Egr-1 residues 335–432). For the sake of simplicity, this construct is hereafter referred to as the Egr-1 zinc-finger protein. The protein was expressed in *Escherichia coli* and purified as described in our previous work (20,22,23). For fluorescence experiments, 33-, 48-, 63-, 88-, 113- and 143-bp probe DNA duplexes with a fluorescein amidite (FAM) attached to the 5′-terminus were prepared using synthetic DNA strands and enzymatic reactions as described (20). The duplexes contain a single Egr-1 target site and share the terminal 33 bp: FAM-AGCGTGGGCGTACCGGTAACTATCGTCTTGAGT-3′ (the Egr-1 target is underlined). Their complete sequences are given in (20). A 28-bp competitor DNA duplex (5′-GTACCGATTGCAGATTCCGAACCTTCAG-3’) containing neither specific nor semi-specific sites was prepared as previously described (20,23).

### Stopped-flow fluorescence assay of the target search kinetics

The target search kinetics was measured at 20°C using an ISS PC-1 spectrofluorometer equipped with an Applied Photophysics RX.2000 stopped-flow device as described (20). In this assay, the time courses of the FAM fluorescence were monitored upon mixing two solutions: (i) the Egr-1 zinc-finger protein, and (ii) FAM-labeled probe DNA and 28-bp nonspecific competitor DNA. Hereafter, the total concentrations of the probe DNA, protein and competitor DNA in the reaction mixture are represented by the symbols *D*_tot_, *P*_tot_, and *C*_tot_, respectively. All experimental and theoretical analyses in this study used the conditions *D*_tot_ « *P*_tot_ « *C*_tot_. Because of these inequalities, the relevant second-order processes occur in a pseudo-first-order manner, which simplifies the kinetic analysis (24). The buffers used were 10 mM Tris·HCl (pH 7.5), 200 nM ZnCl_2_ and 40–400 mM KCl. Apparent pseudo-first-order rate constants *k*_app_ were measured by mono-exponential fitting to the fluorescence time-course data (20). The apparent second-order rate constants *k_a_* for the target association were determined from *k*_app_ data using various protein concentrations *P*_tot_, typically between 20 and 400 nM (see the Supplementary Materials). This range is biologically relevant, because western blot and DNA association data for nuclear extracts (e.g. 18,19) implies that the maximum level of nuclear Egr-1 *in vivo* is of order of 10^−9^–10^−7^ M. To analyze sliding of Egr-1 on DNA, we measured the rate constants *k_a_* using 33-, 48-, 63-, 83-, 113- and 143-bp probe DNA (*D*_tot_ = 2.5 nM) and 28-bp competitor DNA (*C*_tot_ = 2000 nM). To analyze intersegment transfer between two nonspecific sites on distinct DNA duplexes, we measured the rate constants *k_a_* using 113-bp probe DNA (*D*_tot_ = 2.5 nM) and 28-bp competitor DNA at various concentrations (*C*_tot_ = 1000–16 000 nM) (20).

### Analytical expression for the target search kinetics

To analyze the experimental kinetic data, we derived a new general analytical expression for the target search kinetics. The derivation, which is given in the Supplementary Materials, was based on the backward master equations of first-passage probabilities for discrete-state stochastic models (25,26). In our previous study (20), we used the pseudo-first-order rate constant *k*_app_ from fluorescence time-course data along with its analytical form that assumes the conditions *D*_tot_ « *P*_tot_ « *C*_tot_ and quasi-equilibrium for nonspecific protein–DNA interactions prior to the equilibrium of target association. In this study, we used the following analytical form of the second-order rate constant *k_a_* for protein–target association without assuming quasi-equilibrium:

(1) \begin{equation*}
k_a = \frac{S}{{K_{d,N} + (\phi L - S)D_{{\rm tot}} + \phi MC_{{\rm tot}} }} \cdot \frac{1}{{\tau _N }}
\end{equation*}

The parameters in this equation are as follows:

(2) \begin{eqnarray*} &&S = \frac{y(1 + y)(y^{ - L} - y^L )}{ (1 - y)(y^{1 - m} + y^m )(y^{1 + L - m} + y^{m - L} )} \end{eqnarray*}

(3) \begin{equation*}
y = 1 + (1/2)\lambda ^{ - 2} - \sqrt {\lambda ^{ - 2} + (1/4)\lambda ^{ - 4} }
\end{equation*}

(4) \begin{equation*}
\lambda = \sqrt {D_1 \tau _N }.
\end{equation*}

The parameter *λ* is the effective sliding length in units of bp (1,26); *D_1_* is the one-dimensional diffusion coefficient for sliding in units of bp^2^ s^−1^; *K_d,N_* is the equilibrium constant in molar units for each nonspecific site; *L* is the total number of binding sites on the probe DNA; *M* is the total number of binding sites on the competitor DNA; and *m* is the position of the target (located at the *m*th site from the edge). The parameter *ϕ* is the number of possible orientations for each nonspecific site. Due to the structural pseudo-C2 symmetry of double-stranded DNA, *ϕ* = 2 for proteins that bind as a monomer, and *ϕ* = 1 for symmetric dimers. When *ϕ* = 2 is used, the microscopic parameters (e.g. *K_d,N_*, *k*_off_*_,N_*, *k*_IT_*_,N_*) are defined for each orientation (20). The parameter *τ_N_* in Equation (4) is the mean lifetime of a nonspecific complex and is given by

(5) \begin{equation*}
\tau _N = \left[ {k_{{\rm off},N} + k_{{\rm IT},N} \left\{ {\phi LD_{{\rm tot}} + \phi MC_{{\rm tot}} } \right\}} \right]^{ - 1},
\end{equation*}

where *k*_off_*_,N_* is the dissociation rate constant for a nonspecific complex, and *k*_IT_*_,N_* is the second-order rate constant for intersegment transfer (also known as direct transfer) between nonspecific sites on two distinct DNA duplexes (20). Although intersegment transfer can occur between two distant sites on the same molecule if the DNA length is significantly longer than the persistence length (i.e. ∼150 bp), such intra-molecular intersegment transfer is not considered here because only relatively short (<150 bp) DNA duplexes are used in our experiments. The annotation of symbols with the subscript ‘N’ indicates that those symbols represent parameters for ‘nonspecific’ interactions. As explained in the Supplementary Materials, the current analytical expression (Equations (1)–(5)) is more general and simpler than the previous expression, while the two expressions provide numerically equivalent results (e.g. Supplementary Figure S-I). In the Discussion section, we also present a physically clearer description of the *k_a_* constant in terms of the antenna and trapping effects.

### Determination of the parameters for translocation of protein on DNA

The one-dimensional diffusion coefficient *D_1_* and the sliding length *λ* were determined from the length-dependent *k_a_* data by fitting with Equations (1)–(4). The total numbers of binding sites, *L* for the probe DNA and *M* for the competitor DNA, were set to *A* − *B* + 1, where *A* is the DNA length in bp, and *B* is the number of DNA base pairs covered by a protein molecule. Based on structural information about specific and nonspecific DNA complexes of Egr-1 (15,23), *B* = 9 was used, as previously described (20). Based on the sequences of the probe DNA duplexes, the target position *m* = 2 was used. Values of *k_a_* as a function of *L* were given as input data, and the parameters *D_1_* and *λ* were optimized as independent fitting parameters via nonlinear least-squares fitting, in which the sum of the squared differences between observed and calculated *k_a_* values was minimized. In this fitting, *τ_N_* was calculated via Equation (4) with *λ* and *D_1_* being optimized.

The rate constant *k*_IT_*_,N_* for intersegment transfer and the rate constant *k*_off_*_,N_* for dissociation were determined from the dependence of *k_a_* data on competitor DNA concentrations by fitting with Equations (1)–(5). In this calculation, values of *k_a_* as a function of *C*_tot_ were given as input data, and the parameters *k*_IT_*_,N_* and *k*_off_*_,N_* were optimized as independent fitting parameters via nonlinear least-squares fitting. The experimentally obtained *D_1_* coefficient was used in this fitting calculation. Simultaneous fitting to the *L*-dependence and *C*_tot_-dependence data was also conducted using Equations (1)–(5) via optimization of *D_1_*, *k*_IT_*_,N_*, and *k*_off_*_,N_* as the fitting parameters.

These fitting calculations require values of the *K_d,N_* constant for a nonspecific site (see Equation (1)). Values of *K_d,N_* constants used for nonspecific interactions at 40, 60, 80, 110 and 150 mM KCl were 1.4, 3.6, 4.6, 9.6 and 16 μM, respectively, which were obtained from our previous experimental data (20) in conjunction with the counterion condensation theory (27,28). All nonlinear least-squares fitting calculations were performed with the MATLAB software (MathWorks, Inc.). Some examples of our MATLAB scripts for the fitting calculations are given in the Supplementary Materials.

## RESULTS

### Target association kinetics at various ionic strengths

**Figure 1.**
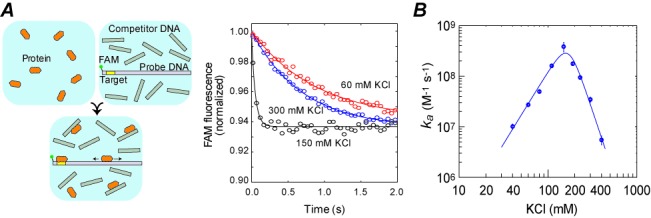
Target search kinetics of the Egr-1 zinc-finger protein as a function of ionic strength. (**A**) Stopped-flow fluorescence assay of the target search kinetics. The fluorescence time-course data shown were obtained using 113-bp probe DNA (*D*_tot_ = 2.5 nM), nonspecific 28-bp DNA (*C*_tot_ = 2000 nM) and protein (*P*_tot_ = 50 nM) at 60, 150 and 300 mM KCl. Concentrations *P*_tot_ and *C*_tot_ were varied in other measurements. (**B**) Apparent second-order rate constants *k_a_* for target association of the Egr-1 zinc-finger protein in the presence of 2000 nM nonspecific 28-bp DNA. The experiment was performed using 113-bp probe DNA at 40, 60, 80, 110, 150, 190, 230, 300 and 400 mM KCl. Values of *k_a_* constant were determined from the pseudo-first-order rate constants for target association at different concentrations of the protein. The solid line shown in the plot is a best-fit curve obtained using Equations (1)–(5) and the counterion condensation theory for the kinetic and thermodynamic parameters (see the main text). The data are shown on a logarithmic scale for each axis. For this panel and for Figures 2 and 3, the error bars represent the standard error of the mean (SEM) estimated from 6–10 replicates. For data points with no error bars, the SEMs were smaller than the size of the symbols.

Using the stopped-flow fluorescence assay, we measured the target search kinetics for the Egr-1 zinc-finger protein. This assay uses three macromolecular components: the probe DNA, protein and nonspecific competitor DNA (Figure 1A). The probe DNA duplexes contain a target site and a FAM moiety that is tethered to a position near the target. The fluorescence from the FAM probe changes upon binding of the protein to the target site (20). Immediately after a solution of the protein is rapidly mixed with a solution containing the probe DNA and competitor DNA, we began monitoring the time courses of the FAM fluorescence intensity (some example data are shown in Figure 1A). The time courses of fluorescence intensity were mono-exponential. The percentage change from initial to final intensities was virtually the same for the time-course data obtained under solution conditions where complete saturation of binding to the target site is expected at equilibrium (e.g. those shown in Figure 1A). A smaller percentage change was observed under conditions where the saturation level at equilibrium is lower (e.g. at 400 mM KCl). Using the time-course data, the pseudo-first-order rate constants in s^−1^ units were determined at various concentrations of protein, from which the apparent second-order rate constant *k_a_* in M^−1^ s^−1^ units was calculated (see Supplementary Figure S-III). Using this assay, we measured the apparent second-order rate constants *k_a_* for target association of Egr-1 with the 113-bp probe DNA in the presence of 2 μM nonspecific 28-bp competitor DNA at 40, 60, 80, 110, 150, 190, 230, 300 and 400 mM KCl. Figure 1B shows a logarithmic plot of the *k_a_* data as a function of KCl concentration. We found that the *k_a_* constant depended strongly on ionic strength in a non-monotonic manner and ranged from 0.055 × 10^8^ to 1.98 × 10^8^ M^−1^ s^−1^. Interestingly, the Egr-1 zinc-finger protein exhibited the fastest target search kinetics at 150 mM KCl. Because Mg^2+^ ions are known to play important roles in some protein–DNA/RNA interactions, we examined the influence of Mg^2+^ ions on the target search kinetics under the same ionic strength. Keeping 0.5 × [K^+^] + 2 × [Mg^2+^] + 0.5 × [Cl^−^] = 150 mM, we measured the target association kinetics as a function of Mg^2+^ concentration in a physiological range (0.5–5 mM). The presence of Mg^2+^ ions caused a slight (up to 23%) increase in the rate constant *k_a_* with the maximum *k_a_* found at [Mg^2+^] = 1 mM (Supplementary Figure S-IV). These results indicate that physiological ionic strength renders an optimal condition for target search by the Egr-1 zinc-finger protein.

### Ionic-strength dependence of sliding

**Figure 2.**
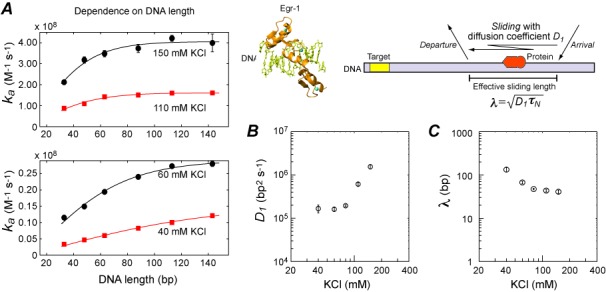
Ionic-strength dependence of sliding. (**A**) Length dependence of *k_a_* constants at 40, 60, 110 and 150 mM. The horizontal axis represents DNA length in bp. Solid lines are the best-fit curves obtained using Equations (1)–(4). The parameter *L*, which represents the number of binding sites on the probe DNA, was set to (DNA length in bp) −8 (see the main text). The one-dimensional diffusion coefficient *D_1_* and the effective sliding length *λ* were optimized in the fitting calculations. *D*_tot_ = 2.5 nM and *C*_tot_ = 2 μM were used in the measurements. (**B**) The one-dimensional diffusion coefficient *D*_1_ for sliding of the Egr-1 zinc-finger protein as a function of KCl concentration. (**C**) The effective sliding length *λ* as a function of ionic strength.

The DNA-length dependence of the target association kinetics provides information on sliding of proteins on DNA (20,29–33). Using the 33-, 48-, 63-, 88-, 113- and 143-bp probe DNA (*D*_tot_ = 2.5 nM) and 28-bp nonspecific competitor DNA (*C*_tot_ = 2000 nM), we measured the apparent rate constants *k_a_* for the Egr-1 zinc-finger protein at various KCl concentrations (Figure 2A). The DNA-length dependence of *k_a_* constants for these probe DNA duplexes was almost linear at 40 mM KCl, but more curved and asymptotic at higher ionic strengths. Based on the length-dependence data, we determined the one-dimensional diffusion coefficient *D*_1_ (in bp^2^ s^−1^; Figure 2B) and the effective sliding length *λ* (in bp; Figure 2C) as a function of KCl concentration. Although some literature assumed that the *D_1_* coefficient for sliding should be independent of ionic strength (11,34–36), we observed significant ionic-strength dependence of the experimental *D_1_* coefficient for the Egr-1 zinc-finger protein (Figure 2B). Our interpretation of this result is given in the Discussion section. We also found that the sliding length *λ* depended strongly on ionic strength: for example, *λ* = 122 ± 2 bp at 40 mM KCl, and *λ* = 38 ± 3 bp at 150 mM KCl. As noted previously (6,20), the DNA-length dependence of the target association kinetics should be an increasing function with an asymptote for lengths > 2*λ*. In fact, such asymptotic length dependence was clearly observed (Figure 2A). At ionic strengths as high as 300 mM KCl, no dependence on length was found for the 33–143 bp DNA duplexes (e.g. see *k_a_* values in Supplementary Figures S-IIIb and c). Although we cannot determine the sliding length *λ* in such a case, the absence of length dependence qualitatively suggests a very short sliding length. At 40–150 mM KCl, the length dependence of *k_a_* constants for the same set of DNA duplexes was strong enough due to 2*λ* being comparable to the DNA lengths used in the experiments (Figure 2A and B).

### Ionic-strength dependence of intersegment transfer and dissociation

**Figure 3.**
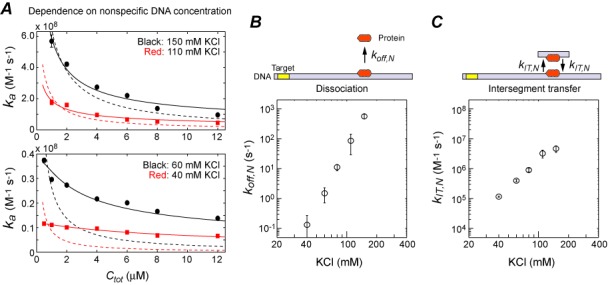
Ionic-strength dependence of the dissociation and intersegment transfer of the Egr-1 zinc-finger protein. (**A**) Dependence of the *k*_app_ constant on the concentration *C*_tot_ of the 28-bp nonspecific DNA. Solid lines are the best-fit curves obtained using a model with intersegment transfer (i.e. Equations (1)–(5)), and dotted lines are the best-fit curves using a model without intersegment transfer (i.e. Equations (1)–(5) with *k*_IT_*_,N_* = 0). (**B**) The rate constant *k*_off_*_,N_* for dissociation from a nonspecific site as a function of KCl concentration. (**C**) The rate constant *k*_IT_*_,N_* for intersegment transfer between two nonspecific DNA molecules as a function of KCl concentration.

Intersegment transfer is the direct translocation of protein from one DNA duplex to another (without going through the intermediary of free protein) via an intermediate in which a protein molecule transiently bridges two DNA duplexes. The DNA concentration dependence of the translocation kinetics allows us to distinguish intersegment transfer from translocation via dissociation and re-association (20,23,37–43). We varied the concentration of the 28-bp nonspecific competitor DNA between 0.5 and 16 μM and measured the rate constant *k_a_* for the association of Egr-1 to the target on the 113-bp probe DNA (Figure 3A). Using the DNA concentration-dependence data, we determined the rate constant *k*_IT_*_,N_* for intersegment transfer between nonspecific sites of two DNA duplexes and the rate constant *k_off,N_* for the dissociation of nonspecific complexes as a function of KCl concentration (Figure 3B and C).

A surprising finding from this analysis was that the ionic-strength dependence of *k*_off_*_,N_* is extremely strong; for example, *k*_off_*_,N_* = 0.20 ± 0.26 s^−1^ at 40 mM KCl and *k*_off_*_,N_* = 920 ± 240 s^−1^ at 150 mM KCl. As shown in Figure 3B, the ionic-strength dependence of *k*_off_*_,N_* clearly obeys the log–log linear relationship in accordance with the kinetic theory by Lohman *et al.* (44), which is based on the counterion condensation arguments (27,28). We found that the rate constant *k*_IT_*_,N_* for intersegment transfer also depended strongly on the ionic strength, but to a lesser degree. Due to this difference, translocation via intersegment transfer is more efficient than translocation via dissociation and re-association at relatively low ionic strengths (i.e. *k*_IT_*_,N_ϕMC*_tot_ > *k*_off_*_,N_*), whereas the latter can be more efficient (i.e. *k*_IT_*_,N_ϕMC*_tot_ < *k*_off_*_,N_*) at higher ionic strengths. In fact, for the *C*_tot_-dependence data at low ionic strengths, the kinetic model with intersegment transfer gave a far better fit than the model without intersegment transfer, whereas the two models gave more similar results for the data at high ionic strengths (Figure 2A). At KCl concentrations significantly higher than 150 mM, translocation via dissociation and re-association governs the *C*_tot_-dependence and conceals the influence of intersegment transfer, which precluded us from further analyses.

## DISCUSSION

### Consideration on the ionic-strength dependent *D_1_* coefficient

Although some previous studies assumed that sliding of proteins on DNA should be virtually independent of ionic strength (11,34–36), our *D_1_* data for Egr-1 (Figure 2B) show significant dependence on ionic strength. Bonnet *et al.* also showed similar results for the restriction enzyme EcoRV (45). One may interpret that ionic-strength dependence of the experimental *D_1_* data is influenced by ‘hopping’ (i.e. short-range correlated translocation via dissociation and re-association (1)). Because our kinetic model does not discriminate between short-range (i.e. hopping) and long-range translocations via dissociation followed by re-association, kinetics of dissociation in these processes are equally represented by the rate constant *k*_off_*_,N_*. The *D_1_* measurement can be significantly affected by hopping if the intrinsic dissociation rate constant for hopping is comparable to the first-order rate constant *k*_sl_*_,N_* for sliding from one site to an adjacent site. To achieve this condition, however, the intrinsic dissociation rate constant for hopping must be >1000-fold larger than the observed dissociation rate constants *k*_off_*_,N_* (see Figures 2B and 3B; note that the *D_1_* coefficient in bp^2^ s^−1^ units is equivalent to *k*_sl_*_,N_* in s^−1^ units (20)). Such extremely rapid dissociation only for hopping seems unrealistic, although we cannot completely exclude this possibility. Thus, it is unlikely that the ionic-strength dependence of the *D_1_* data for Egr-1 arises from the influence of hopping.

A more reasonable interpretation of the ionic-strength dependent *D_1_* data is that energy barriers of electrostatic nature are involved in the sliding process. This interpretation is consistent with results of some previous studies. For example, coarse-grained molecular dynamics simulations of the DNA search by proteins also indicated significant dependence of the *D_1_* coefficient on ionic strengths (23,46,47). Besides, if sliding is barrierless diffusion (e.g. ‘movement on an isopotential surface’ as speculated by Winter *et al.* three decades ago (11)), then the *D_1_* coefficient should be a relatively simple function of the hydrodynamic radius, as is the case for three-dimensional diffusion in solutions. Despite this expectation, data available in the literature for proteins of different sizes show that *D_1_* is virtually independent of molecular size (e.g. 20,35,36,45,48,49). Furthermore, many proteins exhibit sliding on DNA far slower than expected from the Stokes–Einstein relation (as reviewed in (5)). These observations and our ionic-strength dependent *D_1_* data can be explained if sliding involves electrostatic energy barriers, which could be relevant to breakage of ion pairs between protein and DNA (50).

### Egr-1's kinetic properties are well suited for efficient scanning of DNA *in vivo*

Our kinetic data show that the Egr-1 zinc-finger protein exhibits its optimal search efficiency at the physiological ionic strength (i.e. 150 mM KCl). In addition, two other findings in our current study suggest that Egr-1 has characteristics that enable efficient scanning of DNA in the nuclei. First, the effective sliding length for the Egr-1 zinc-finger protein at 150 mM KCl seems to be suitable for scanning accessible regions of chromosomal DNA. According to theoretical work by Mirny *et al.* (6), proteins can scan DNA most efficiently when 2*λ* is comparable to the lengths of the linker DNA between nucleosomes. In human cells, linker lengths are widely distributed with an average of ∼56 bp (13). Because our data indicate that 2*λ* is comparable to these lengths, the sliding and dissociation properties seem suitable for Egr-1 to scan linker DNA *in vivo*. Second, our data also indicate that the intersegment transfer of Egr-1 is very efficient at physiological ionic strength. The previous study using the coarse-grained molecular dynamics simulations suggested that Egr-1 can conduct intersegment transfer between two DNA duplexes separated by 60 Å (23). Because this separation corresponds to the distance between two DNA ends of a nucleosome particle (51), intersegment transfer could allow Egr-1 to efficiently bypass nucleosome particles and continuously scan DNA (20,23). Thus, our data collectively suggest that the kinetic properties of Egr-1 are well suited to efficiently scan DNA *in vivo*.

### Influences of the antenna and trapping effects and intersegment transfer

Our data show that the target search by the Egr-1 zinc-finger protein is fastest at physiological ionic strength. What is the origin for the optimal kinetic efficiency at physiological ionic strength? To offer a theoretical explanation, we provide a general expression for the kinetics of target location by DNA-binding proteins. This is done by transforming Equation (1) into the following form, which shows how the apparent rate constant *k_a_* for the target association is related to the intrinsic association rate constant *k*_on_*_,N_* ( = *k*_off_*_,N_* /*K_d,N_*) of each site:

(6) \begin{equation*}
k_a = \rho \eta Sk_{{\rm on},N}.
\end{equation*}

The parameters *ρ* and *η* in this equation are as follows:

(7) \begin{equation*}
\rho = \frac{{K_{d,N} }}{{K_{d,N} + (\phi L - S)D_{{\rm tot}} + \phi MC_{{\rm tot}} }}
\end{equation*}

(8) \begin{equation*}
\eta = 1 + \frac{{k_{{\rm IT},N} \left( {\phi LD_{{\rm tot}} + \phi MC_{{\rm tot}} } \right)}}{{k_{{\rm off},N} }}.
\end{equation*}

Equation (6) quantitatively explains how the target association kinetics is influenced by the antenna effect, the trapping effect, and intersegment transfer, which are represented by the parameters *S*, *ρ*, and *η*, respectively. The parameter *S* given by Equation (2) corresponds to the effective size of the antenna. Nonspecific sites near the target on the same DNA serve as an antenna and make the target association *S*-fold faster. Because this is equivalent to *S* sites serving as the target, the term *ϕL* – *S* in Equation (7) corresponds to the number of nonspecific sites outside the antenna on the target-containing DNA duplex, and (*ϕL* – *S*)*D*_tot_ + *ϕMC*_tot_ corresponds to the net overall concentration of nonspecific sites. Therefore, the parameter *ρ* represents the fraction of protein molecules that are not trapped by any nonspecific sites during the target search process. If (*ϕL* – *S*)*D*_tot_ « *ϕMC*_tot_ (which is usually satisfied), then the parameter *ρ* becomes independent of *S*, and the expression becomes equivalent to the expression given by Esadze and Iwahara under the assumption of quasi-equilibrium for nonspecific interactions (20) (see the Supplementary Materials). If the target position is near the middle of the DNA duplex (i.e. *m* ≈ *L*/2), the parameter *S* (Equation (2)) becomes independent of *m* and can be reduced to

(9) \begin{equation*}
S \approx 2\lambda \tanh \left( {\frac{L}{{2\lambda }}} \right),
\end{equation*}

which corresponds to the expression for the antenna effect given in previous literature (1,6) (see Supplementary Figure S-II). Equation (8) shows that intersegment transfer considerably enhances the target search kinetics (i.e. *η* » 1) when and only when the pseudo-first-order rate constant for intersegment transfer is substantially larger than the dissociation rate constant of nonspecific complexes. As indicated by Equation (6), the ratio of *k_a_* to *k_on,N_* is given by *ρηS*, in which *ρ* represents an attenuation factor (0 < *ρ* < 1) due to the trapping effect, *η* is an enhancement factor (*η* > 1) due to intersegment transfer, and *S* is an enhancement factor (1 < *S* < 2*λ*) due to the antenna effect.

### Optimal target search by Egr-1 at physiological ionic strengths

**Figure 4.**
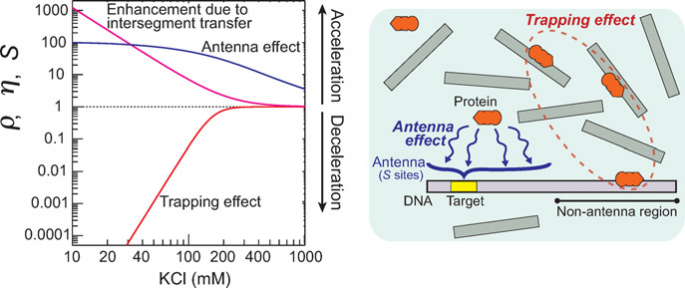
The attenuation factor *ρ* due to the trapping effect (red), the enhancement factor *η* due to intersegment transfer (magenta) and the enhancement factor *S* due to the antenna effect (blue) for the target association kinetics of the Egr-1 zinc-finger protein. These parameters were calculated from the current experimental data on the rate constants *k*_off_*_,N_* and *k*_IT_*_,N_*, the one-dimensional diffusion coefficient *D_1_*, and the dissociation constant *K_d,N_* for the Egr-1 zinc-finger protein along with Equations (2), (7) and (8). The following conditions were used: *D*_tot_ = 2.5 nM; *L* = 105 sites; *C*_tot_ = 2000 nM; *M* = 20 sites. Ionic-strength dependence represented by ${\rm log}k = a{\rm log}[{\rm KCl}] + b$ was assumed for *k*_off_*_,N_*, *k*_IT_*_,N_*, *D_1_* and *K_d,N_*, and the parameters *a* and *b* were calculated from the current experimental data.

Based on the above analytical expression of *k_a_*, we can now explain the ionic-strength dependence of the target search kinetics in terms of the antenna effect, the trapping effect and intersegment transfer. Using the current experimental data, we calculated the parameters *ρ*, *η* and *S* for the Egr-1 zinc-finger protein as shown in Figure 4. The parameters *S* and *η* are decreasing functions of ionic strength, and the antenna effect and intersegment transfer substantially accelerate the target search kinetics at low ionic strengths. In contrast, the parameter *ρ* is an increasing function of ionic strength, and the trapping effect substantially decelerates the target search kinetics at low ionic strengths. As previously described, the rate constants for electrostatically assisted macromolecular association (such as *k*_on_*_,N_*) are typically a decreasing function of ionic strength (52,53). The rate constant *k_a_* as the product *ρηSk*_on_*_,N_* (Equation (6)) is therefore maximized at a particular ionic strength as shown in Figure 1B. Our data indicate that the Egr-1 zinc-finger protein achieves its optimal kinetic efficiency at physiological ionic strength through the compromise between the positive and negative impacts of nonspecific interactions with DNA.

### CONCLUSIONS

In this study, we have demonstrated the optimal search efficiency of the Egr-1 zinc-finger protein at physiological ionic strength and explained the ionic-strength dependence of the target search kinetics in terms of the antenna and trapping effects and intersegment transfer. Our results suggest that Egr-1's kinetic properties in the sliding, dissociation and intersegment transfer processes are well suited for efficient scanning of DNA *in vivo*. This study provides insight into how the inducible transcription factor Egr-1 rapidly activates the genes in response to cellular stimuli such as synaptic signals and vascular injury. This work also provides a theory that quantitatively explains the interplay between the antenna and trapping effects and intersegment transfer in the target DNA search process. A deeper understanding of this interplay could be helpful in the engineering of transcription factors and DNA repair/modifying enzymes. For example, our theory predicts that protein engineering that simply increases the affinity for DNA will enhance the trapping effect and possibly result in slow target location. Because Egr-1 (Zif268) has been used as a scaffold for zinc-finger technology for artificial gene control, we expect that current and future studies of DNA scanning by Egr-1 at the molecular and atomic levels may facilitate the kinetic improvement of artificial zinc-finger proteins.

## SUPPLEMENTARY DATA

Supplementary Data are available at NAR Online.

SUPPLEMENTARY DATA
