# Supplementary material for: Positive and negative impacts of nonspecific sites during target location by a sequence-specific DNA-binding protein: origin of the optimal search at physiological ionic strength
Source: Nucleic Acids Res. 2014 May 16;42(11):7039–46. doi: 10.1093/nar/gku418 (PMC4066804; doi:10.1093/nar/gku418)
Supplement: SUPPLEMENTARY DATA [file supp_42_11_7039__index.html]

Positive and negative impacts of nonspecific sites during target location by a sequence-specific DNA-binding protein: origin of the optimal search at physiological ionic strength — Positive and negative impacts of nonspecific sites during target location by a sequence-specific DNA-binding protein: origin of the optimal search at physiological ionic strength — SUPPLEMENTARY DATA 

# Positive and negative impacts of nonspecific sites during target location by a sequence-specific DNA-binding protein: origin of the optimal search at physiological ionic strength

## SUPPLEMENTARY DATA

**Files in this Data Supplement:**

- SUPPLEMENTARY DATA
