# Supplementary material for: Positive and negative impacts of nonspecific sites during target location by a sequence-specific DNA-binding protein: origin of the optimal search at physiological ionic strength
Source: Nucleic Acids Res. 2014 May 16;42(11):7039–46. doi: 10.1093/nar/gku418 (PMC4066804; doi:10.1093/nar/gku418)
Supplement: SUPPLEMENTARY DATA [file supp_gku418_nar-00565-h-2014-File006.pdf]

SUPPLEMENTARY MATERIAL:

Positive and negative impacts of nonspecific sites during target location by a sequence-specific DNA-binding protein: Origin of the optimal search at physiological ionic strength

Alexandre Esadze, Catherine A. Kemme, Anatoly B. Kolomeisky, and Junji Iwahara

A. Theoretical model for protein search for targets on DNA in the presence of competing nonspecific DNA and intersegment transfer: The derivations of Eqs. 1-8

To simplify calculations we develop a single-protein search model that can be easily adjusted for any concentration of proteins and DNA. In our model, we consider some volume that has d identical probe DNA duplexes, each having L discrete binding sites, with a specific target at the site m ($1\leq m\leq L$). Also in the system there are c competing DNA duplexes (M sites each) that do not have specific target sites. There is one protein molecule that can bind to any site on any DNA with the rate k_{on}, or it can be unbound from DNA to the surrounding solution with the rate k_{off}. When bound to the DNA the protein might slide along the chain to the neighboring sites with the rate u. In addition, the bound protein molecule can jump onto any other site on any DNA with the rate r in the process known as intersegment transfer. We associate the time to find the target with a first-passage times, a theoretical concept that is well developed for various stochastic processes in Chemistry, Physics and Biology (25). At t=0 the protein molecule starts in the solution. Let us define a function $F_n(t)$ as a probability to reach the target for the first time at time t if the protein molecule starts at the site n ($1\leq n\leq L$) of any probe DNA molecules at t=0. We also define $F_0(t)$ as a first-passage probability to reach the target if the protein molecule starts in the solution (state 0). In addition, $F_c(t)$ defines the probability to reach the target starting from any site on the competing DNA molecules (state c). Then the mean-first passage time to reach the target from the solution is given by

$$\tau = \int_0^\infty tF_0(t)dt. \qquad \text{(s1)}$$

Dynamic evolution of first-passage probabilities is governed by a set of backward master equations (25):

$$\frac{dF_n(t)}{dt} = u[F_{n-1}(t) + F_{n+1}(t)] + crMF_c(t) + dr\sum_{n=1}^{L} F_n(t) + k_{off}F_0(t) - (2u+crM+drL+k_{off})F_n(t); \qquad \text{(s2)}$$

$$\frac{dF_0(t)}{dt} = dk_{on}\sum_{n=1}^{L} F_n(t) + ck_{on}MF_c(t) - (dk_{on}L + ck_{on}M)F_0(t); \qquad \text{(s3)}$$

$$\frac{dF_c(t)}{dt} = dr\sum_{n=1}^{L} F_n(t) + k_{off}F_0(t) - \big(drL + k_{off}\big)F_c(t). \qquad \text{(s4)}$$

These expressions must be also supplemented by initial conditions that require $F_m(t)=\delta(t)$ and $F_{i\neq m}(t=0)=0$ with $i=n$, c or 0. The physical meaning of initial conditions is the following: if the protein molecule starts at the specific target then the search time is equal to zero, otherwise it is nonzero for any other initial position.

The most convenient way of analyzing these backward master equations is to employ Laplace transformations of first-passage probability functions,

$$\widetilde{F_n(s)} = \int_0^\infty e^{-st} F_n(t)dt. \quad \text{(s5)}$$

Then the average time to find the target can be easily found from the corresponding Laplace form,

$$\tau = -\frac{d\widetilde{F_0}}{ds}|_{s=0}. \quad \text{(s6)}$$

Using Laplace transformations the backward master equations can be written as a set of simpler algebraic expressions,

$$\left(s + 2u + cMr + drL + k_{off}\right)\widetilde{F_n} = u\left(\widetilde{F_{n+1}} + \widetilde{F_{n-1}}\right) + cMr\widetilde{F_c}dr\sum_{n=1}^{L}\widetilde{F_n} + k_{off}\widetilde{F_0}; \quad \text{(s7)}$$

$$[s + k_{on}(dL + cM)]\widetilde{F_0} = cMk_{on}\widetilde{F_c} + dk_{on}\sum_{n=1}^{L}\widetilde{F_n}\ ; \quad \text{(s8)}$$

$$\left(s + Ldr + k_{off}\right)\widetilde{F_c} = dr\sum_{n=1}^{L}\widetilde{F_n} + k_{off}\widetilde{F_0}. \quad \text{(s9)}$$

These equations are solved by assuming that the general solution has the following form,

$$\widetilde{F_n} = Ay^n + B, \quad \text{(s10)}$$

where unknown parameters *A*, *B* and *y* are determined from substituting this expression into Eqs. s8-s10 and also using initial and boundary conditions. One can derive then,

$$y(s) = \frac{\left(s+2u+cMr+drL+k_{off}\right) - \sqrt{\left(s+2u+cMr+drL+k_{off}\right)^2 - 4u^2}}{2u}, \quad \text{(s11)}$$

and

$$B = \frac{[sr+k_{on}\left(k_{off}+dLr+cMr\right)]\widetilde{F_0}}{k_{on}\left(s+k_{off}+dLr+cMr\right)}. \quad \text{(s12)}$$

Now from Eqs. s8-s10 it can be shown that

$$\widetilde{F_0} = \frac{k_{on}d\left(s+k_{off}+dLr+cMr\right)S}{s\left(s+k_{on}dL+k_{off}\right)+sk_{on}cM+dS[k_{on}\left(k_{off}+dLr+cMr\right)+sr]}, \quad \text{(s13)}$$

where the new auxiliary function *S*(*s*) was introduced via

$$S(s) = \frac{y(s)\{1+y(s)\}\{y(s)^{-L}+y(s)^L\}}{\{1-y(s)\}\{y(s)^{1-m}+y(s)^m\}\{y^{1+L-m}+y^{m-L}\}}. \quad \text{(s14)}$$

This analysis allows us to obtain the exact analytical expression for the average time to reach the target by using Eqs. s6 and s14:

$$\tau = \frac{(L-S)k_{on}d+k_{off}+k_{on}cM+drS}{k_{on}dS\left(k_{off}+dLr+cMr\right)}, \quad \text{(s15)}$$

where we used $S \equiv S(s=0)$.In the case of no intersegment transfer and without competing non-specific DNA this result reduces to the one obtained earlier by Veksler and Kolomeisky (26). The expression for the average search time for a single protein can be easily transformed for any concentration of proteins and DNA, leading to the following form of the apparent second-order rate constant k_a for the protein – target association (using the corresponding bulk notations),

$$k_a = \frac{S\{k_{off,N}+k_{IT,N}(\phi MC_{tot}+\phi LD_{tot})\}}{K_{d,N}+(\phi L-S)D_{tot}+\phi MC_{tot}+\left(\frac{k_{IT,N}}{k_{on,N}}\right)SD_{tot}} \tag{s16}$$

$$= \frac{S}{K_{d,N}+(\phi L-S)D_{tot}+\phi MC_{tot}+\left(\frac{k_{IT,N}}{k_{on,N}}\right)SD_{tot}} \cdot \frac{1}{\tau_N} \tag{s17}$$

$$= \rho_e \eta S k_{on,N} \tag{s18}$$

The symbols used in Eqs. s16-s18 are defined in the main text. The parameter ρ_e corresponds to the attenuation factor due to the trapping effect and is given by:

$$\rho_e = \frac{K_{d,N}}{K_{d,N}+(\phi L-S)D_{tot}+\phi MC_{tot}+(\frac{k_{IT,N}}{k_{on,N}})SD_{tot}} \tag{s19}$$

It is important to note that the term $(k_{IT,N} / k_{on,N})SD_{tot}$ is negligible in the denominator of Eqs. s16, s17, and s19 under the conditions of $D_{tot} << P_{tot} << C_{tot}$, which are satisfied under our experimental conditions. It should be also noted that *in vivo* environments involve a vast number of nonspecific DNA segments and only a small number of target-containing DNA segments. Eqs. 1, 6, and 7 in the main text are obtained by neglecting the $(k_{IT,N} / k_{on,N})SD_{tot}$ term. This simplification is very convenient for two reasons. First, it gives a more practical expression for analyzing experimental data. In particular, Eqs. 1-5 together with experimentally obtained $K_{d,N}$ permit determination of the 1-D diffusion coefficient D_1 and the sliding length λ from the DNA length-dependent k_a data without requiring any information of $k_{IT,N}$, $k_{off,N}$, and $k_{on,N}$. This is not possible with Eq. s16, since it requires the knowledge of $k_{IT,N}$ and $k_{on,N}$. Second, by using Eq. 7 instead of Eq. s19, the physical meaning of the enhancement factor S becomes more apparent, as described in the main text. Eqs. 6-8 provide a clear explanation on the relationship between k_a and $k_{on,N}$ in terms of the antenna and trapping effects and intersegment transfer.

B. Comparison with the previous expression for target association kinetics

The following expression for the target association kinetics under the pseudo-first-order conditions due to $D_{tot} << P_{tot} << C_{tot}$ was obtained in our previous work (20) assuming the quasi-equilibrium of nonspecific protein-DNA interactions prior to the equilibrium of the system:

$$k_a = \frac{1}{D_{tot}}\left(\frac{f_P}{T_P} + \frac{f_{CP}}{T_{CP}}\right) \tag{s20}$$

The parameters f_P and f_{CP} represent the fractions of proteins in the free state and in the state of nonspecific complexes with competitor DNA, respectively; and T_P and T_{CP} represent the mean search times for a free protein molecule and for a protein initially bound to competitor DNA, respectively. They are given by:

$$f_P = \frac{K_{d,N}}{K_{d,N}+\phi M C_{tot}} \quad \text{(s21)}$$

$$f_{CP} = \frac{\phi M C_{tot}}{K_{d,N}+\phi M C_{tot}} \quad \text{(s22)}$$

$$T_P = \{L k_{out} + (L-S) k_{in,P}\}/(k_{out} k_{in,P} S) \quad \text{(s23)}$$

$$T_{CP} = \{L k_{out} + (L-S) k_{in,CP}\}/(k_{out} k_{in,CP} S), \quad \text{(s24)}$$

in which the relevant kinetic parameters are as follows:

$$k_{in,P} = L k_{on,N} D_{tot} \quad \text{(s25)}$$

$$k_{in,CP} = L k_{IT,N} D_{tot} \quad \text{(s26)}$$

$$k_{out,P} = \tau_N^{-1} = k_{off,N} + \phi M k_{IT,N} C_{tot} \; . \quad \text{(s27)}$$

The new expression for the apparent rate constant for target association (i.e., Eqs. 1-8 in the main text and Eqs. s16-s18) is more general because it does not assume the quasi-equilibrium of the nonspecific protein-DNA interactions prior to the equilibrium of the target association.

Despite the difference in appearance, the previous and new expressions provide virtually the same values of the rate constant k_a, at least for our experimental conditions. Figure S-I shows comparison of the previous and new expression. Values calculated with the two expressions agree well because the inequalities $D_{tot} << P_{tot} << C_{tot}$, which are assumed to be correct for both approaches, make the assumption of quasi-equilibrium valid. However, the new analytical expression is superior because it is simpler and it provides physical explanations of the impact of the antenna and trapping effects and intersegment transfer in the form of Eq. 6 in the main text.

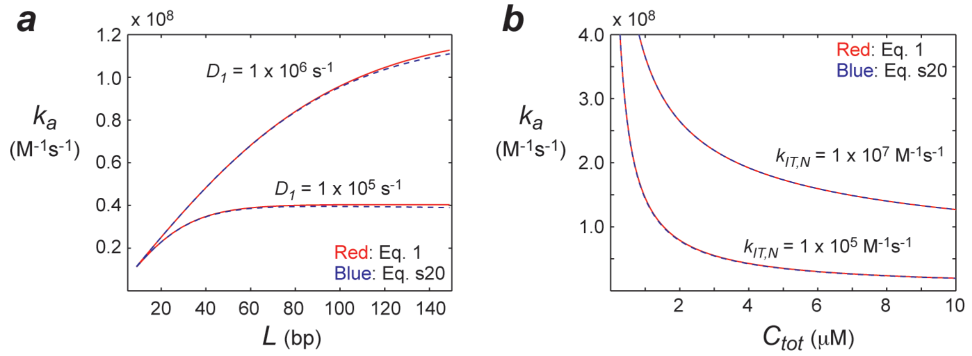

Figure S-I. Comparison of the rate constants k_a calculated with Eq. 1 and with Eq. s20. (***a***) DNA length-dependence. (***b***) Competitor concentration-dependence for the probe DNA. For both pannels, the following parameters were used: D_{tot} = 2 nM; $K_{d,N}$ = 5 μM; $k_{off,N}$ = 100 s^{-1}; ϕ = 2; M = 20 bp; and m = 2. In addition, $k_{IT,N} = 10^5$ M^{-1} s^{-1} was used for the panel *a*, and L = 100 bp and $D_1 = 5 \times 10^5$ s^{-1} were used for the panel *b*.

C. The parameter *S* as the enhancement factor due to the antenna effect

The parameter S (Eq. 2 in the main text), which was originally introduced by Veksler and Kolomeisky (26), represents the antenna effect as explained in the Discussion section of the main text. In fact, as shown in Figure S-IIa, the parameter S given by Eq. 2 agrees well with the previous expression (Eq. 9)(1,6) for the antenna effect with a target site near the middle (i.e., $m \approx L/2$). If the target is near the edge of DNA, the antenna effect becomes weaker, requiring the use of Eq. 2. Figure S-IIb shows an example with m = 1.

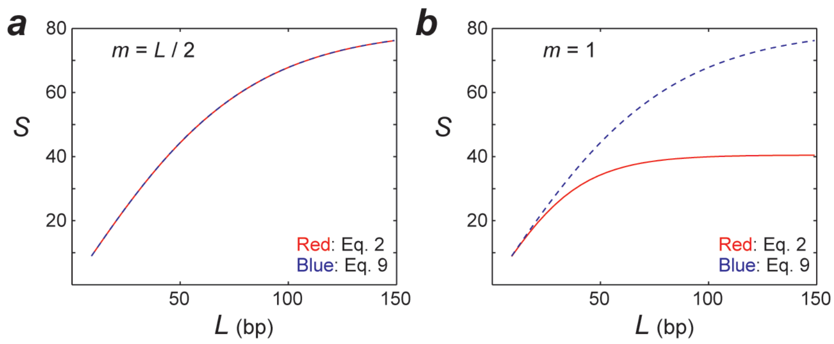

Figure S-II. The parameter S calculated with Eq. 2 and Eq. 9 in the main text. Values of S were calculated as a function of the total number of sites (L) on DNA. The target site is at the mth position from the edge of DNA (***a***, $m = L / 2$; and ***b***, $m = 1$). The sliding length $\lambda = 40$ bp was used.

D. Importance of protein-concentration dependence in k_a analysis

As described previously, the apparent pseudo-first-order rate constant k_{app} from mono-exponential fitting to the stopped-flow fluorescence time-course data is given by (20):

$$k_{app} = k_a P_{tot}(1 + \frac{K_{d,S}}{f_P P_{tot}}) \quad , \qquad \text{(s28)}$$

where $K_{d,S}$ is the dissociation constant for the specific complex with the target. The $K_{d,S} / f_P P_{tot}$ term in Eq. s28 is due to the contribution of the backward process. Under the conditions where saturation of binding to the target occurs at equilibrium, this second term is negligible and k_{app} is directly proportional to P_{tot}. In such a case, k_a can be accurately determined from the k_{app} measured at a single concentration of protein. However, if information about the affinity $K_{d,S}$ for the target or about the saturation level of the binding to the target is unavailable, it is important to determine k_a from k_{app} values measured at multiple concentrations of protein. The protein-concentration dependence should be linear with the slope corresponding to k_a and the intercept corresponding to the contribution of the backward process (i.e., $k_a K_{d,S}/f_P$). Figure S-III shows some examples of protein concentration- dependent k_{app} data. In the case of the Egr-1 zinc-finger protein, the intercept was significant only at 400 mM KCl. For lower concentrations of KCl ($\leq$ 300 mM), the rate constant k_{app} was directly proportional to the total concentration of protein (P_{tot}) due to higher affinities that render $K_{d,S} / f_P P_{tot} << 1$.

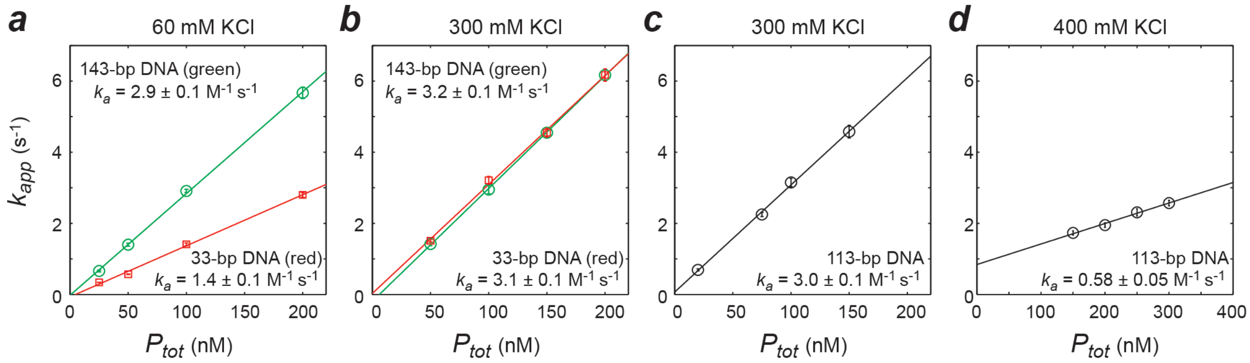

Figure S-III. Protein concentration-dependence of the apparent pseudo-first-order rate constant k_{app} measured for the Egr-1 zinc-finger protein at 60, 300, and 400 mM KCl. FAM-labeled probe DNA (D_{tot} = 2.5 nM; length indicated) and the 28-bp nonspecific competitor DNA (C_{tot} = 2,000 nM) were used. Values of the apparent second-order rate constant k_a determined as the slope are also indicated.

E. Mg^{2+}-concentration dependence

Because divalent ions are known to play important roles in many protein-nucleic acids interactions, we examined impact of Mg^{2+} on the target DNA search by the Egr-1 zinc-finger protein. Since the k_a constant strongly depends on ionic strength as shown in Figure 1B, we used buffers of the same ionic strength containing distinct amounts of Mg^{2+} ions (0 – 5 mM), keeping $0.5 \times [K^+] + 2 \times [Mg^{2+}] + 0.5 \times [Cl^-] = 150$ mM. Using the FAM-labeled 113-bp probe DNA (D_{tot} = 2.5 nM) and the 28-bp nonspecific DNA (C_{tot} = 8,000 nM) in these buffers, we measured the k_a constant as a function of Mg^{2+} concentration in a physiological range. We found that the impact of Mg^{2+} ions on the target DNA search by the Egr-1 zinc-finger protein was relatively minor, as shown in Figure S-IV.

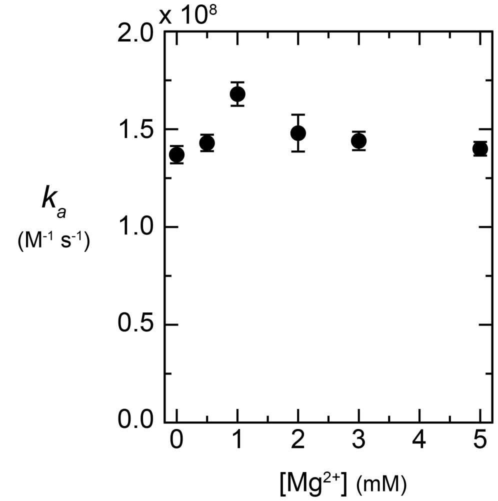

Figure S-IV. Mg^{2+}-concentration dependence of the apparent second-order rate constant k_a measured for the Egr-1 zinc-finger protein. The concentration of the 28-bp competitor DNA was 8,000 nM. The buffers used were 10 mM Tris•HCl (pH7.5), 200 nM $ZnCl_2$, 135 – 150 mM KCl, and 0 – 5 mM $MgCl_2$, and the overall ionic strength was kept constant. Each error bar represents a standard error of the mean (SEM) for 7 – 10 replicates.

F. Source codes of MATLAB scripts/functions for analysis of translocation kinetics

F-1. MATLAB script "FitLDepend.m"

```
%%%%%%%%%%%%%%%%%%%%%%%%%%%%%%%%%%%%%%%%%%%%%%%%%%%%%%%%%%%%%%%%%%%%%%%%%%%%%%%%
% FitLDepend: Script to determine the parameters D1 and lambda for sliding %
%                                                                          %
% Programmed by Junji Iwahara (j.iwahara@utmb.edu)                         %
%                                                                          %
% This script requires the function "calLDepKa.m" in the same directory.   %
%%%%%%%%%%%%%%%%%%%%%%%%%%%%%%%%%%%%%%%%%%%%%%%%%%%%%%%%%%%%%%%%%%%%%%%%%%%%%%%%
clc; clear; close all;
global m Dtot Ctot M Kdn phi

%%%%%%%%%%%%%%%%%%%%%%%%%%%%%%%%%%%%%%%%%%%%%
% Geometrical Information (Setting by User) %
%%%%%%%%%%%%%%%%%%%%%%%%%%%%%%%%%%%%%%%%%%%%%

% Position of the target on probe DNA (position from the edge)
m = 2;
```

```
% Length of competitor DNA (in bp)
lcomp = 28;
% Length of each nonspecific site (in bp)
clnb  = 9;
% Number of orientations for each nonspecific site
phi = 2; % 2 for monomeric protein; 1 for symmetric dimer

%%%%%%%%%%%%%%%%%%%%%%%%%%%%%%%%%%%%%%%%%%%%%%%%%%%%%
% Experimental Information & Data (Setting by User) %
%%%%%%%%%%%%%%%%%%%%%%%%%%%%%%%%%%%%%%%%%%%%%%%%%%%%%

% Total concentration of probe DNA in M (Dtot)
Dtot = 2.5*10^(-9);
% Total concentration of competitor DNA in M (Ctot)
Ctot = 2000.0*10^(-9);
% DNA lengths (in bp)
DNAlengths = [  33   48   63   88  113  143];
% Apparent second-order rate constants ka in M-1 s-1 for target association
kas        = [2.44 3.50 4.00 4.58 4.96 4.98]*10.0^7;% (EXPERIMENTAL DATA)
% Standard deviation
errKas     = [0.16 0.36 0.38 0.40 0.36 0.44]*10.0^7;
% # of replicates
nexps      = [   8    9    9    9   10    7];
% Standard error of mean (SEM)
sems        = errKas ./ sqrt(nexps);
% Dissociation constant (in M) for a nonspecific site (EXPERIMENTAL DATA)
Kdn = 4.6*10^(-6);

%%%%%%%%%%%%%%%%%%%%%%%%%%%%%%%%%%%%%%%%%%%%%%%%%%%%%%
% Initial values for nonlinear least-squares fitting %
%%%%%%%%%%%%%%%%%%%%%%%%%%%%%%%%%%%%%%%%%%%%%%%%%%%%%%
lambdaGuess = 30.0 ; % Try different values if fitting does not work
D1Guess  = 1.0*10^4; %

%%%%%%%%%%%%%%%%%%%%%%%%%%%%%%%%%%%%%%%%%%%%%%%%%%%%%%%%%%%%%%%%
%              NO MODIFICATION NEEDED BELOW THIS               %
%%%%%%%%%%%%%%%%%%%%%%%%%%%%%%%%%%%%%%%%%%%%%%%%%%%%%%%%%%%%%%%%

% Number of sites on competitor DNA
M = lcomp - clnb + 1;
% Number of sites on probe DNA
Ls = DNAlengths - clnb + 1;

%Nonlinear least-squares fitting
opts =statset('MaxIter',4000);
[Kfit,resid,jacob] = nlinfit(Ls,kas,@calLDepKa,[lambdaGuess D1Guess],opts);
cl=68; % confidence limit (for one sigma)
ke = nlparci(Kfit, resid,'jacobian',jacob,'alpha',(100-cl)/100.0);
fprintf('Lambda: %7.4f +/- %e bp\n',abs(Kfit(1)),(ke(1,2)-ke(1,1))/2);
fprintf('D1: %e +/- %e bp2 s-1\n',  abs(Kfit(2)),(ke(2,2)-ke(2,1))/2);
lfs = (Ls(1)-2):(Ls(length(Ls))+5);
fitCurve = calLDepKa(Kfit,lfs);
%Plot (Horizontal axis: DNA length in bp)
plot(lfs+clnb-1,fitCurve)
hold on
errorbar(DNAlengths,kas,sems,'o')
hold off
ylim([0 max(kas)*1.15])
```

F-2. MATLAB function “calLDepKa.m”

```
function k = calLDepKa( K, Ls )
%calLDepKa:
% Calculation of the second-order rate constant ka as a function of L
%
%-Arguments-
% K:  array of fitting parameters (lambda and D1)
% Ls: array of numbers of sites on the probe DNA
%
global m Dtot Ctot M Kdn phi
lamb = abs(K(1));
D1 = abs(K(2));
L = Ls;
% Based on Eq. 4
tauN = (lamb^2)/D1;
% Eq. 3
y = 1.0 + 0.5/(lamb^2) - sqrt(1.0/(lamb^2) + 0.25 /(lamb^4));
% Eq. 2
s= y .* (1 + y) .* (y.^(-L) - y.^L) ...
    ./ (1 - y) ./ (y.^(1 - m) + y.^m) ./ (y.^(1 + L - m) + y.^(m - L));
% Eq. 1
k=s ./ tauN ./ (Kdn + (L .* phi - s) .* Dtot + M .* Ctot .*phi);
end
```

F-3. MATLAB script “FitCtotDepend.m”

```
%%%%%%%%%%%%%%%%%%%%%%%%%%%%%%%%%%%%%%%%%%%%%%%%%%%%%%%%%%%%%%%%%%%%%%%%%%%%%%%
% FitCtotDepend: Script to determine the rate constants koffN and kitN     %
%                                                                          %
% Programmed by Junji Iwahara (j.iwahara@utmb.edu)                         %
%                                                                          %
% This script requires the function "calCtotDepKa.m" in the same directory.%
%%%%%%%%%%%%%%%%%%%%%%%%%%%%%%%%%%%%%%%%%%%%%%%%%%%%%%%%%%%%%%%%%%%%%%%%%%%%%%%
clc; clear; close all;
global m Dtot M Kdn Lp D1 phi swIT

%%%%%%%%%%%%%%%%%%%%%%%%%%%%%%%%%%%%%%%%%%%%%
% Geometrical information (Setting by User) %
%%%%%%%%%%%%%%%%%%%%%%%%%%%%%%%%%%%%%%%%%%%%%

% Probe DNA length (in bp)
DNAlength  = 113;
% Position of the target on probe DNA (position from the edge)
m = 2;
% Competitor DNA length(in bp)
lcomp = 28;
% Length of each nonspecific site (in bp)
clnb  = 9;
% Number of orientations for each nonspecific site
phi = 2; % 2 for monomeric protein; 1 for symmetric dimer

%%%%%%%%%%%%%%%%%%%%%%%%%%%%%%%%%%%%%%%%%%%%%%%%%%%%%
% Experimental Information & data (Setting by User) %
%%%%%%%%%%%%%%%%%%%%%%%%%%%%%%%%%%%%%%%%%%%%%%%%%%%%%

% Total concentration of probe DNA in M (Dtot)
```

```
Dtot = 2.5*10^(-9);
% Total competitor concentrations in M (Ctot)
Ctots    = [ 0.5  1.0  2.0  4.0  6.0  8.0]*10^(-6);
% Apparent second-order rate constants ka in M-1 s-1 for target association
kas      = [8.96 7.04 4.96 4.42 3.04 3.46]*10.0^7;%(EXPERIMENTAL DATA)
% Standard deviations
errKas   = [1.10 0.70 0.36 0.52 0.30 0.26]*10.0^7;
% # of replicates
nexps    = [   8    8   10    8    9    9];
% Standard error of mean (SEM)
sems     = errKas ./ sqrt(nexps);
% Dissociation constant (in M) for a nonspecific site (EXPERIMENTAL DATA)
Kdn = 4.6*10^(-6);
% 1-D diffusion constant in bp2 s-1 for sliding (EXPERIMENTAL DATA)
D1 = 1.94*10^5;  % From fitting to length-dependence kapp data

%%%%%%%%%%%%%%%%%%%%%%%%%%%%%%%%%%%%%%%%%%%%%%%%%%%%%
% Initial values for nonlinear least-squares fitting %
%%%%%%%%%%%%%%%%%%%%%%%%%%%%%%%%%%%%%%%%%%%%%%%%%%%%%
koffNguess = 1.0    ;  % Try different values if fitting does not work
kitNguess  = 1.0*10^5; %

%%%%%%%%%%%%%%%%%%%%%%%%%%%%%%%%%%%%%%%%%%%%%%%%%%%%%%%%%%%%%%%%
%              NO MODIFICATION NEEDED BELOW THIS               %
%%%%%%%%%%%%%%%%%%%%%%%%%%%%%%%%%%%%%%%%%%%%%%%%%%%%%%%%%%%%%%%%

% Number of sites on probe DNA
Lp = DNAlength - clnb + 1;
% Number of sites on competitor DNA
M = lcomp - clnb + 1;

%Nonlinear least-squares fitting (Intersegment transfer (IT) model)
swIT = 1;
options = statset('MaxIter',8000);
[Kfit,resid,jacobian] = nlinfit(Ctots,kas,@calCtotDepKa,...
    [koffNguess kitNguess],options);
cl=68; % confidence level (for one sigma)
ke = nlparci(Kfit, resid,'jacobian',jacobian,'alpha',(100-cl)/100.0);
fprintf(' kitN  = %e +/- %e s-1\n',abs(Kfit(2)),(ke(2,2)-ke(2,1))/2);
fprintf(' koffN = %e +/- %e s-1\n',abs(Kfit(1)),(ke(1,2)-ke(1,1))/2);
fprintf(' From koff and Kdn \n konN  =');
fprintf(' %e +/- %e M-1 s-1\n',abs(Kfit(1))/Kdn,(ke(1,2)-ke(1,1))/2/Kdn);
cs = (Ctots(1)*0.75):10^(-8):(Ctots(length(Ctots))*1.05);
fitCurveWithIT = calCtotDepKa(Kfit,cs);

%Nonlinear least-squares fitting (No IT model)
swIT = 0;
[KfitNoIT,residNoIT] = nlinfit(Ctots,kas,@calCtotDepKa,...
    koffNguess,options);
fitCurveWithoutIT = calCtotDepKa(KfitNoIT,cs);
%Plot (solid, with IT; dotted, without IT)
plot(cs,fitCurveWithIT,'r-',cs,fitCurveWithoutIT,'g:')
hold on
errorbar(Ctots,kas,sems,'bo')
hold off
```

F-4. MATLAB function “calCtotDepKa.m”

```
function k = calCtotDepKa( K, Ctots )
%calCtotDepKa:
% Calculation of the rate constant ka as function of Ctot
%
%-Arguments-
% K: array of fitting parameters (koffN and kitN)
% Ctots: array of Ctot values
%
global m Dtot M Kdn phi D1 Lp swIT
koffN = abs(K(1));
if (swIT == 0)
    kitN = 0;           % For the model without intersegment transfer
else
    kitN = abs(K(2)); % For the model with intersegment transfer
end
L = Lp;
% Eq. 5
tauN = 1.0 ./ (koffN + kitN .*(M .* Ctots .*phi + L .* Dtot .*phi));
% Eq. 4
lamb = sqrt(D1 .* tauN);
% Eq. 3
y = 1.0 + 0.5 ./ (lamb .^2) - sqrt(1.0 ./(lamb .^2) + 0.25 ./(lamb .^4));
% Eq. 2
s= y .* (1 + y) .* (y.^(-L) - y.^L) ...
    ./ (1 - y) ./ (y.^(1 - m) + y.^m) ./ (y.^(1 + L - m) + y.^(m - L));
% Eq. 1
k=s ./ tauN ./ (Kdn + (L .* phi - s) .* Dtot + M .* Ctots .*phi);
end
```
